# Skin reflectance changes in Kenyan neonates during the first month of life: an observational study

**DOI:** 10.1038/s41390-025-04079-w

**Published:** 2025-05-09

**Authors:** Seth Bokser, Priscillah Koech, Hillary Bosuben, Anne Gaichiumia, Atsushi Miwa, Anthony Wanyoro

**Affiliations:** 1 Department of Pediatrics, University of California San Francisco, San Francisco, CA USA; 2 Kenyatta University, Nairobi, Kenya; 3 Kiambu County Level 5 Regional Hospital, Kiambu County, Kenya; 4 Vital Sensing Group, Konica Minolta Inc., Chiyoda, Tokyo Japan

## Abstract

- The study documents significant changes in skin pigmentation among Kenyan newborns over their first month of life, with significant decreases in reflectance at wavelengths critical for transcutaneous diagnostic devices.
- This research provides the first quantitative documentation of optical-light characteristics in African newborn skin using Individual Typology Angle (ITA), demonstrating significant darkening as infant skin matures.
- By establishing baseline ITA values and developmental changes in African newborns, this study creates an evidence-based framework for designing future inclusive clinical trials for transcutaneous diagnostic devices that account for both skin tone diversity and age-related changes.

## Introduction

Transcutaneous diagnostic devices are widely used in neonatal care. The American Academy of Pediatrics (AAP) advises universal newborn screening for hyperbilirubinemia, commonly performed with transcutaneous bilirubin (TcB) meters.^1^ Both the U.S. Centers for Disease Control and AAP recommend universal cyanotic congenital heart disease screening using pulse oximeters.^2^

Although widely adopted, these transcutaneous devices have faced renewed scrutiny regarding their diagnostic accuracy in patients with darker skin tones, driven by increasing awareness of racial and ethnic disparities in healthcare.^3^ In vitro studies have demonstrated falsely lower TcB readings with darker modeled skin tones,^4^ while human population studies have, more often, demonstrated falsely elevated TcB readings in neonates with racial identities associated with darker skin tone.^5,6^ Other studies have examined biochemical development and color development in lighter skin tone newborns.^7^ To our knowledge, this is the first study to document developmental changes in skin tone among African newborns with darker skin pigmentation.

The U.S. Food and Drug Administration (FDA) and the International Council for Harmonization (ICH) have both emphasized the importance of including diverse patient populations, particularly across different skin tones, in trials assessing transcutaneous diagnostic devices.^8,9^ Our study can provide these future trials with foundational insights into the optical characteristics of newborn skin correlating not only with skin tone but also developmental age of the pediatrics patient.

## Methods

### Study design, participants, and assessments

This prospective cohort study was conducted at the Level 5 Regional Hospital in Kiambu County, Kenya, with approval from the Institutional Review Boards at the University of California, San Francisco and Kenyatta University (Nairobi, Kenya). The purpose of this study was to measure and assess changes in skin tone and reflectance in Kenyan newborns using a full-optical spectrophotometer (CM-700d /Konica Minolta Inc.) and a targeted transcutaneous bilirubin meter (JM-105 /Konica Minolta Inc.).

Eighty newborns, each meeting inclusion criteria—including weight of more than 1.75 kg and estimated gestational age (GA) of over 34 weeks based on obstetrical history or physical examination—were enrolled in the study during their first week of life (referred to as “Visit 1”). Of the 80 newborns, 40 returned to the hospital for a follow-up spectrophotometry at one-month of age. Six of these newborns were excluded from the “Visit 2” follow-on analysis due to issues with spectrophotometry technique, including ambient light contamination and incomplete data capture. Therefore, data from 34 newborns were included in the cohort follow-on component analysis.

### Assessments and statistical analysis

We measured skin tone by calculating Individual Typology Angle (ITA), which is based on skin reflectance properties in the CIE L*a*b* color space.^10^ ITA = arctan((L* − 50)/b*) × (180/π), where L* represents lightness and b* represents yellow/blue chromaticity. ITA has become the gold standard for assessing human skin tone over historically referenced Fitzpatrick and Monk scales, due to its high accuracy, reproducibility, and reduced operator bias.^11^ ITA also correlates closely with the melanin index for African patient populations.^12^

ITA values were derived from one-time measurements obtained using a CM-700d spectrophotometer on the skin located over the sternum, below the nipple line, and above the xiphoid process in all subjects. The CM-700d is the highest quality handheld spectrophotometer with a standard reproducibility rate of σΔEab 0.04. (ΔEab is a metric that quantifies the difference between two colors.) A ΔE*ab value of 1.0 is often considered the threshold at which the human eye can detect a color difference under ideal conditions. CM-700d derived ITA values were categorized for this study based on previously published work correlating ITA with other established skin tone scales.^13^ Study data were collected and managed using secure REDCap data capture tools hosted at the University of California, San Francisco.

Descriptive statistics are presented as means with ranges. Differences between neonates included and excluded from follow-up analysis were assessed using independent *t*-tests. For longitudinal comparisons between Visit 1 and Visit 2 measurements, paired *t*-tests were performed. Simple linear regression was used to examine whether the timing of initial measurements (ranging from 2 to 188 hours of life) influenced Visit 1 ITA values. Statistical analyses were performed using R version 4.4.1 (2024-06-14) within the RStudio Integrated Development Environment (IDE) Build 547 (2023-07-06).

## Results

Baseline cohort characteristics are described in Table 1. For newborns included in the follow-on analysis, the mean age of measurement was 67 hours for Visit 1 and 36 days for Visit 2. There were no significant baseline differences between subjects included and excluded from the Visit 2 follow-on analysis (see Table 1 and limitations). Regression analysis within Visit 1 of the relationship between age at first measurement (2–188 h of life) and ITA values showed no significant correlation within any group (full cohort: *r* = 0.01, *p* = 0.939; excluded neonates: *r* = −0.00, *p* = 0.990; included neonates: *r* = 0.03, *p* = 0.861). These findings indicate that the timing of initial measurements within the first week of life did not systematically affect our study findings on developmental changes in ITA values.

**Table 1:**
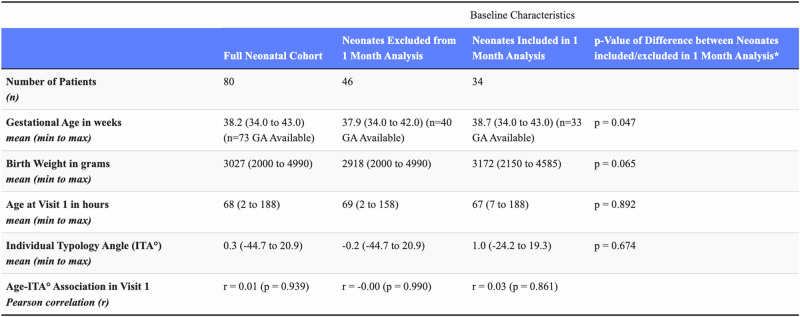
Kenyan neonatal cohort characteristics.

Data are presented as mean (min to max) unless otherwise. Age-ITA correlations show no relationship between age at measurement and ITA values within Visit 1.

Table 2 illustrates the shift in skin tone for Kenyan newborns over the first month of life. Most newborns followed (21 of 34) had ITAs in category 3 during the first week of life, whereas less than 10 per cent (3 out of 34 subjects) remained in that lighter tone category after 1 month of normal development. At one-month of age, nearly half of our Kenyan cohort (16 of 34) had matured into the darkest ITA Skin Type Categories: Category 5 (ITA −50 to < −25) and Category 6 (ITA < −50).

**Table 2:**
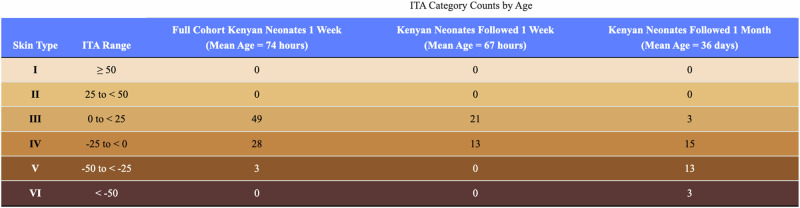
ITA category counts at 1 week and 1 month of age.

Fitzpatrick colors applied for visualization.

Table 3 illustrates that over the first month of life, the optical appearance of skin became darker (decrease in L*), less red (decrease in a*), and less yellow (decrease in b*). The mean ITA decreased by an average of 24 degrees over the first month of life from 1 degree (min −24°, max 19°) to −23° (min −62°, max 6°). Cutaneous reflectance decreased significantly over the first month of life at clinically significant wavelengths of 450 (2.52%), 460 (2.58%), 550 (4.31%), 560 (4.39%), and 660 nm (10.83%) (all *p* < 0.001).

**Table 3:**
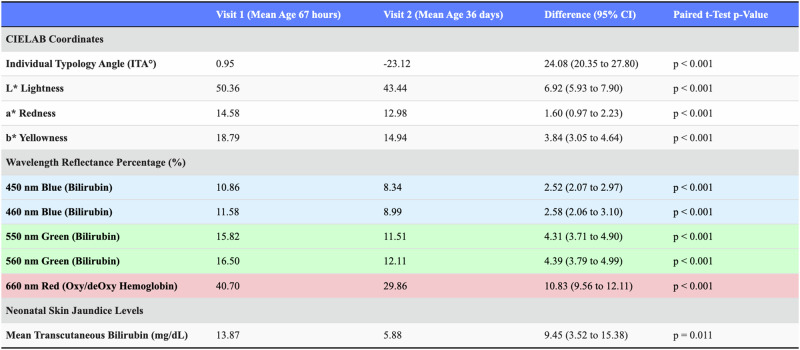
Sepctrophotometry mean CIE Lab* coordinates and mean wavelength relative reflectance.

Wavelength reflectance is provided as mean relative values.

Table 4 visually represents ITA changes for each infant from Visit 1 to Visit 2, clearly demonstrating the consistent pattern of darkening skin tone across the cohort as well as individual variation.

**Table 4:**
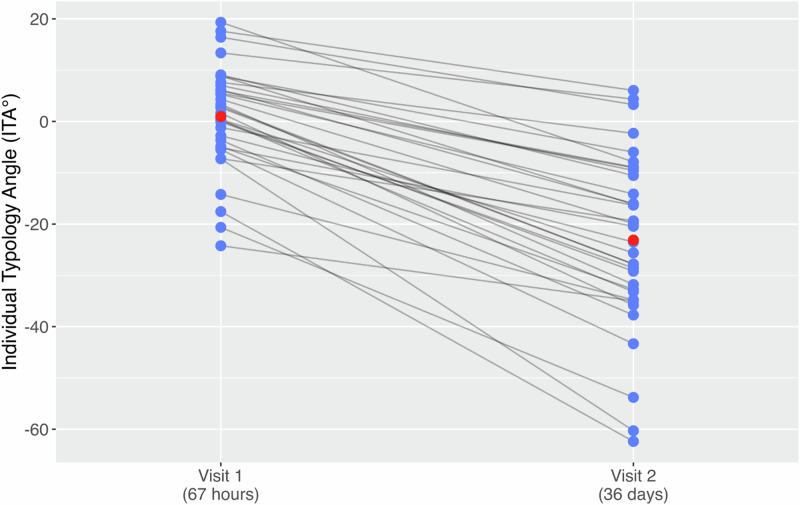
Visualization of ITA changes over first month of life.

Blue dots represent individual participant measurements. Gray lines connecting measurements from the same participant. Red points show mean ITA values for the followed neonatal at each timepoint.

## Discussion

Our study demonstrates significant physiologic maturation toward darker skin pigmentation in Kenyan newborns over the first month of life. Changes in reflectance for darker pigmented Kenyan newborns are significant at the clinical wavelengths used by transcutaneous bilirubin meters to detect bilirubin (450, 460, 550, and 560 nm) and used by many pulse oximeters to detect oxy/deoxy hemoglobin (660 nm). Table 3 also shows that newborn bilirubin levels decreased as expected between Visit 1 and Visit 2 (*p* = 0.011). Analysis of Individual Typology Angle (ITA) values between visits revealed that the change in skin darkness (L* component) was primarily responsible for the observed decrease in ITA, with minimal contribution from the yellow-blue (b*) component. When isolating the impact of each component, changing only the L* value (while keeping b* constant) accounted for ~80–85% of the total ITA decrease, while changing only the b* value produced negligible change in ITA (0.3°). This finding, coupled with the significant decreases in reflectance despite declining bilirubin levels (which would theoretically increase reflectance in the blue-green spectrum, particularly around 450–460 nm), suggests that melanin-driven skin maturation has a stronger effect on optical properties than changes in bilirubin levels.

Documentation of Kenyan newborn ITA and CIE L*a*b* coordinates, as shown in Tables 2 and 3, lays a critical foundation for designing trials that build diverse neonatal cohorts to evaluate transcutaneous diagnostic devices. Additionally, our findings suggest that neonatal and pediatric trials should account for age-related changes in skin pigmentation when validating and improving transcutaneous diagnostics. If there exists systemic error for transcutaneous devices associated with darker pigmented newborn patients, our reflectance data suggest the error may emerge or worsen with increasing skin maturity over the first month of life.

Our findings on skin tone maturation are consistent with earlier work by Visscher et al., who documented skin maturation changes in premature infants. Unlike previous studies, our research specifically quantifies changes in skin reflectance at clinically relevant wavelengths for darker skin tone African late preterm and term infants.

Limitations of our study include finite sample size (34 patients in follow-on analysis) born in one region of Kenya. Additional studies are needed to validate these findings in other darker skin tone populations. Furthermore, reliance on a single-point skin measurement on the sternum is applicable to the transcutaneous bilirubin meter being studied but may limit generalizability of our results for pulse oximeters, which are often applied on the hands and feet. Another limitation, shown in Table 1, is that mean gestational age differed slightly between the neonates included and excluded from the one-month follow-on analysis. For participants included in the follow-on analysis, the mean estimated gestational age was 38.7 weeks, slightly higher than the 37.9 weeks for those excluded (*p* = 0.047). Importantly, all other baseline variables—most importantly initial ITAs—were no different, affirming our belief that this minor difference in an often-estimated baseline clinical measure of gestational age would not impact our results or conclusions. Additionally, our study was limited by not performing concurrent subjective Monk or Fitzpatrick scale assessments— though we did estimate Fitzpatrick skin type digital color-coding for visual reference in Table 2. Pediatrics studies comparing these subjective scoring systems with gold standard spectrophotometric measurements could help validate these simpler assessment tools for study recruitment and future use in resource-limited settings. Finally, while we used Krishnapriya et al.’s categorization bins for ITAs, other classification ranges exist in the literature. For example, Wilkes et al. have advocated for universal categorization of ITA that is correlated with melanin index.

## Conclusion

In conclusion, our study provides the first quantitative documentation of optical-light characteristics in neonatal African infant skin, underscoring that both skin tone and cutaneous reflectance change significantly during the first month of life. In our Kenyan cohort, neonatal skin darkness increased—primarily driven by changes in the L* component—and reflectance decreased at wavelengths used diagnostically by transcutaneous bilirubin meters and pulse oximeters. By establishing baseline ITA values and developmental changes in African newborns, this research creates an important evidence base for future inclusive clinical trials. As research on the accuracy of transcutaneous diagnostics in pediatrics progresses, it is essential to consider ITA skin tone, infant age, and expected maturational changes in neonatal skin.
